# Spatiotemporal analysis of atmospheric aerosols in African environments using MERRA-2 data (1980–2024): Impacts on climate extremes

**DOI:** 10.1016/j.isci.2025.112995

**Published:** 2025-06-25

**Authors:** Daniel O. Omokpariola

**Affiliations:** 1 Environmental Chemistry and Toxicology Research Unit, Department of Pure and Industrial Chemistry, Nnamdi Azikiwe University, P.M.B 5025, Awka, Anambra State, Nigeria; 2 Department of Production and Technical, Office Chérifien des Phosphates (OCP) Africa Fertilizers Nigeria Limited, Abuja, Nigeria

**Keywords:** Atmospheric science, Atmospheric chemistry, Atmospheric observation

## Abstract

This study investigates atmospheric aerosol trends and their climatic impacts across Africa from 1980 to 2024 using MERRA-2 reanalysis data. It reveals rising concentrations of organic and black carbon, dust, and PM_2_._5_, largely driven by industrialization and urbanization. These aerosols influence climate extremes by altering radiation balance and cloud dynamics, contributing to increased heatwave frequency, temperature rise, and precipitation variability. The study employs spatiotemporal analysis and predictive modeling to assess aerosol-climate interactions and their implications for human health, agriculture, and water resources. Findings highlight consistent warming, worsening drought conditions, and elevated public health risks, particularly in urban and arid regions. This work underscores the need for targeted adaptation strategies and air quality management to mitigate the socio-environmental impacts of climate change in Africa.

## Highlights

• Aerosol levels increased sharply due to urbanization and industrial emissions
• PM₂.₅ and dust levels linked to rising health risks in different African cities
• Heatwaves increased in frequency and intensity across Africa since 1980
• Climate extremes correlate strongly with aerosol and PM concentration trends

## Graphical abstract

**Figure:**
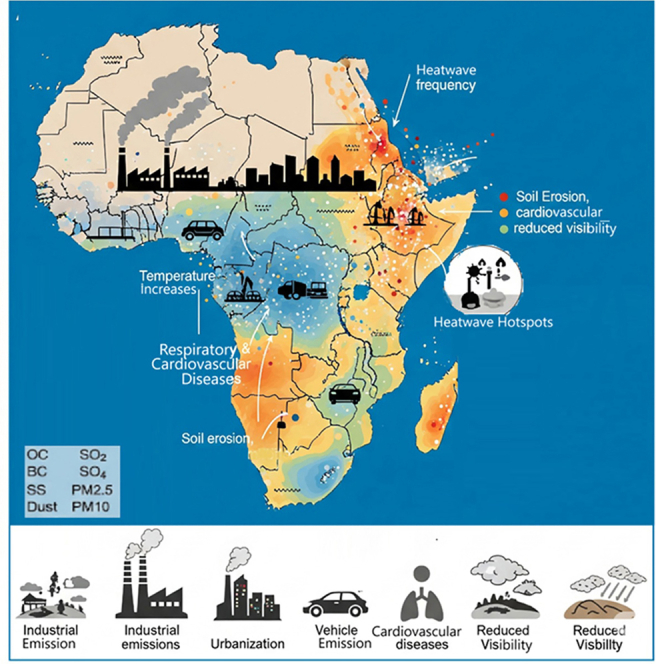

## Introduction

The arid and non-arid regions of Africa are facing unprecedented environmental and climatic challenges, characterized by low precipitation, extreme flooding, drought, sparse vegetation, and extreme temperatures; thus, threatening the livelihoods of millions of people.^1^^,^^2^^,^^3^ Climate extremes, such as prolonged droughts, intense heatwaves, and sporadic flash floods, exacerbate the already fragile ecosystems, leading to severe degradation of soil and water resources, loss of biodiversity, and increased spread of invasive species.^4^^,^^5^^,^^6^^,^^7^ Atmospheric aerosols, which include particulate matter (PM), play a significant role in influencing the climate and environmental conditions in these regions,^8^^,^^9^^,^^10^ yet their spatiotemporal dynamics in these areas remain poorly understood.

Atmospheric aerosols in Africa originate from various natural and anthropogenic sources, including desert dust, biomass burning, industrial activities, and transportation. These aerosols interact with solar radiation, clouds, and atmospheric circulation, influencing temperature, precipitation, and weather patterns.^11^^,^^12^ However, the complex relationships between aerosols, climate, and environment in Africa are not yet fully understood, particularly over several decades.^13^^,^^14^^,^^15^

Extreme weather events in Africa are significantly influenced by aerosol dynamics, particularly through the transport of Saharan dust and the implications of stratospheric aerosol geoengineering (SAI).^16^^,^^17^^,^^18^^,^^19^ The Saharan air layer, which carries substantial mineral dust across the Atlantic, has been linked to extreme weather patterns, as evidenced by events like the June 2015 dust storm, characterized by high aerosol optical depth and intensified atmospheric circulation, which affected radiation balance and weather systems in the region.^16^ Furthermore, studies on SAI indicate that while it may mitigate temperature increases, it could also lead to complex changes in precipitation patterns, with potential increases in annual rainfall but decreases in heavy precipitation events, particularly in the Sahel and Gulf of Guinea regions.^17^^,^^20^ In Africa, mineral dust plays a significant role in aerosol-cloud interaction (ACI) by acting as cloud condensation nuclei (CCN) and ice-nucleating particles (INP), which influence cloud formation and properties, affecting precipitation patterns and cloud albedo.^21^ Biomass burning aerosols also contribute to cloud formation and can modify cloud properties, leading to changes in precipitation and radiation balance.^22^ Aerosol-radiation interaction (ARI) involves aerosols directly scattering and absorbing solar radiation, leading to cooling or warming of the atmosphere depending on the aerosol type.^23^ Dust aerosols generally absorb solar radiation, causing atmospheric warming and surface cooling,^24^ and aerosols can alter the radiative properties of clouds, affecting the Earth’s energy balance.^22^ The mechanism of ACI and ARI varies by region and season. For example, in West Africa, the interaction between dust and clouds is crucial during the West African Monsoon,^20^^,^^21^ while biomass burning aerosols have a more pronounced effect on radiation in southern Africa.^22^

Harr et al.^15^ examined the extreme Saharan dust storm of 2015, highlighting high dust emissions and transport mechanisms that led to significant radiative impacts over the Atlantic. Obahoundje et al.^16^ investigate the influence of SAI on temperature and precipitation extremes in Africa, finding that SAI could reduce warming but alter precipitation patterns, with increased rainfall over the Sahel and Eastern Africa and decreased rainfall over Southern Africa and the Guinea Coast. Alamou et al.^20^ assessed SAI impacts on extreme precipitation and temperature in West Africa, noting mixed changes in precipitation extremes and the influence of sea surface temperature anomalies. Pu and Jin^25^ analyze the record-breaking trans-Atlantic African dust plume of June 2020, attributing it to enhanced dust emissions and atmospheric circulation extremes. Patel et al.^26^ explore SAI’s potential impact on temperature and precipitation extremes in South Africa, highlighting that SAI could significantly reduce the frequency of hot temperature extremes and increase cold temperature extremes, potentially leading to over-cooling in some regions. Huang et al.^27^ investigate the relationship between African aerosols and precipitation variability using satellite data, showing that high aerosol concentrations suppress precipitation. Rodríguez and López-Darias^28^ discuss the northward expansion of extreme Saharan dust events over the Atlantic and Europe, suggesting increased frequency and intensity can change atmospheric circulation patterns. Rushingabigwi et al.^29^ examined the influence of dust and black carbon on clouds in Africa, highlighting significant alterations in cloud properties, affecting cloud formation, precipitation, and radiative forcing. Dagan and Eytan^30^ explore the role of absorbing aerosols in enhancing extreme precipitation events, suggesting that absorbing aerosols could play a significant role in driving extreme weather events, particularly in regions with high aerosol concentrations.

Having assessed the literature, there is a lack of comprehensive understanding of atmospheric aerosol properties and their impacts on climate extremes in arid Africa, which has significant consequences. These include inadequate prediction and preparedness for climate-related disasters, such as droughts and heatwaves, leading to food insecurity, water scarcity, and loss of human life.^23^^,^^31^ Additionally, there is insufficient implementation of effective adaptation and mitigation strategies, exacerbating the vulnerability of these regions to environmental degradation and climate change.^32^^,^^33^^,^^34^ Limited development of sustainable land management practices leads to soil degradation, desertification, and loss of biodiversity.^35^^,^^36^^,^^37^ Inadequate protection of human health, particularly in vulnerable populations, such as children, the elderly, and those with pre-existing medical conditions, who are disproportionately affected by air pollution and climate extremes, is also a major concern.^38^^,^^39^^,^^40^^,^^41^ The interplay between atmospheric aerosols and climate extremes in arid regions of Africa remains underexplored, particularly in terms of their spatiotemporal dynamics and the subsequent effects on ecosystems.^31^^,^^32^ There is a pressing need to understand how variations in aerosol concentrations, driven by both natural and anthropogenic sources, influence climate extremes, and contribute to environmental degradation.

### Study area

**Figure 1:**
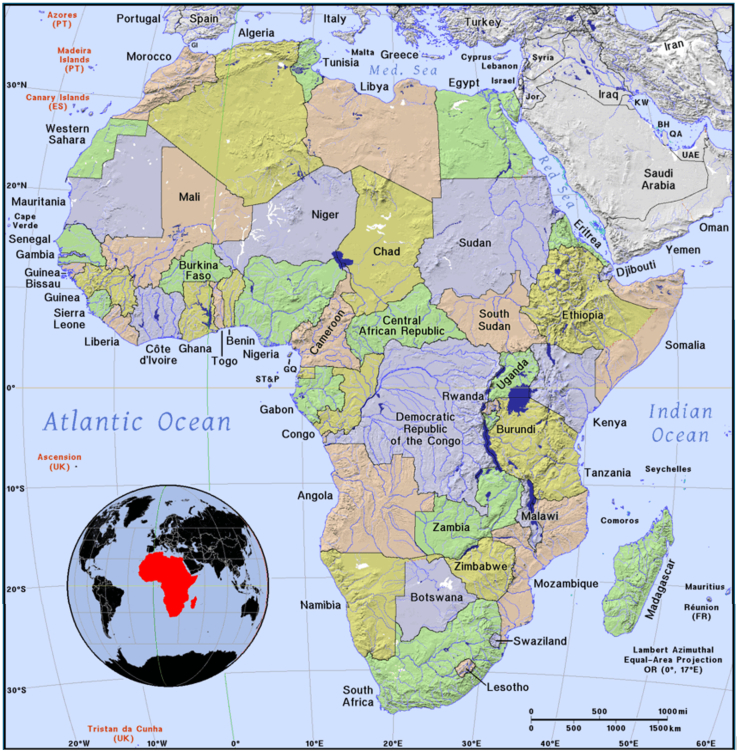
Topography Map of Africa (Retrieved from Macky^42^)

Africa covers approximately 30.37 million square kilometers, making it the second-largest continent by land area (Figure 1). Africa’s vast size encompasses diverse climates, landscapes, and ecosystems’, ranging from deserts and savannas to rainforests and mountains, as politically, it is divided into 5 regions with a total of 54 countries having 7 islands and 47 incontinent countries, respectively.^43^^,^^44^ The climate ranges from arid deserts in the north to tropical rainforests in the central region, as the Sahara Desert dominates the northern region, characterized by extreme temperatures (>40°C) and minimal rainfall (<15%), while the Sahel, a semi-arid region south of the Sahara, experiences more moderate conditions with seasonal rainfall with central Africa is home to lush rainforests with high humidity and consistent rainfall, while Southern Africa features a mix of savannas and semi-arid regions.^45^ Climate change exacerbates issues such as increased desertification in the north and more intense flooding in the southern regions.^45^ The industrialization matrices are uneven, with advanced industries in South Africa and emerging sectors in countries like Nigeria and Kenya that are concentrated in urban areas, leading to increased economic activity, such as manufacturing, mining, and oil extraction are key industries, impacting both the environment and local communities.^46^^,^^47^ The landscape of Africa is incredibly diverse, from the Sahara’s vast dunes to the Great Rift Valley’s dramatic escarpments with increasing urbanization, mining, and agriculture reshaping landscapes, and also, deforestation in tropical areas and land degradation in arid zones.^48^

### Concept of MERRA-2

The MERRA-2 (Modern-Era Retrospective Analysis for Research and Applications, Version 2), a comprehensive reanalysis dataset provided by NASA’s Global Modeling and Assimilation Office (GMAO) and accessible through NASA GIOVANNI (Geospatial Interactive Online Visualization and Analysis Infrastructure) offers atmospheric parameters from various satellite and ground monitoring instruments, including various aerosol components with the dataset spanning from 1980 to the present with high temporal (hourly, daily, seasonal, and annual) and spatial (0.5° × 0.625° latitude-longitude grid, offering a valuable resource for analyzing the long-term trends and impacts of aerosols on climate extremes and ecosystem degradation in African environments.^49^^,^^50^^,^^51^ This study aims to fill these knowledge gaps by leveraging MERRA-2 data to perform a detailed spatiotemporal analysis of atmospheric aerosols in African environments over 45 years (1980–2024). The research will focus on determining the concentrations and impacts of PM_1_, PM_2_._5_, and PM_10_ derived from key aerosol components such as sulfur dioxide (SO_2_), sulfate (SO_4_), organic carbon (OC), black carbon (BC), dust, and sea salt (SS), and evaluating their role in climate extremes (temperature, humidity, heatwave, and precipitation patterns). The study will develop predictive statistical models using advanced classification processes and data-driven approaches for managing the impacts of aerosols and climate extremes in Africa. This study will provide a comprehensive understanding of the spatiotemporal dynamics of atmospheric aerosols in African environments and their impacts on climate extremes. The findings will offer valuable insights for policymakers, environmentalists, and stakeholders, aiding in the development of effective strategies for managing air quality and mitigating the adverse effects of climate change in the African region.

## Result

### Annual assessment of atmospheric particulate precursors

**Figure 2:**
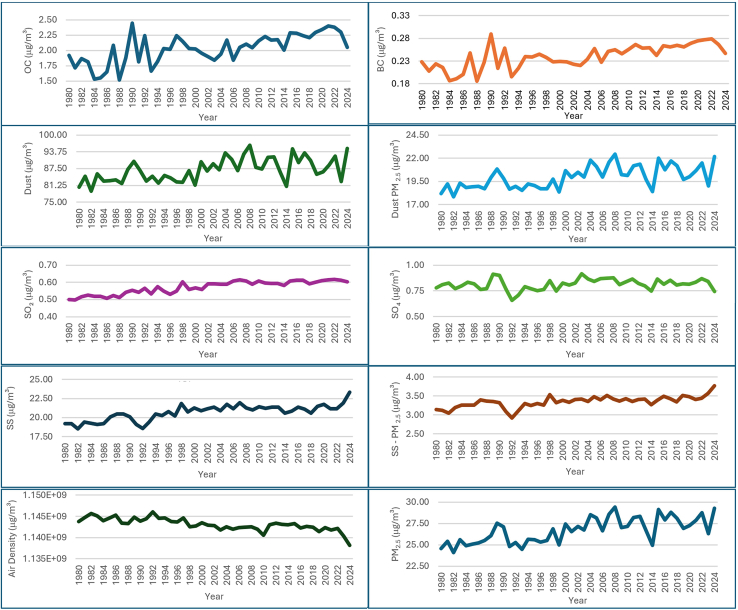
Annual trend chart of atmospheric aerosols (OC: organic carbon; BC: black carbon; SO_2_: sulfur dioxide; SO_4_: sulfate; SS: sea salt; PM2.5: particulate matter 2.5)

Figure 2 and Table S1 shows the trend data spans from 1980 to 2024 and annual concentrations of various atmospheric aerosols and their constituents with OC and black carbon (BC) concentrations exhibiting fluctuations over the years, with OC ranging from 1.52 to 2.44 μg/m^3^ and BC from 0.19 to 0.29 μg/m^3^ with noticeable increase in both OC and BC concentrations toward the later years, indicating a potential rise in combustion-related activities, which is similar to Hamsha^52^ research in Jordan. The dust and dust PM_2_._5_ concentrations remain relatively high throughout the years, with a range of 79.1–96.2 μg/m^3^; and 17.8 to 22.4 μg/m^3^ that reflects the PM component of dust, which has significant health implications to human health in Africa. The sulfur dioxide (SO_2_) showed slight variations ranging from 0.50–0.62 μg/m^3^, with a general increase toward the later years, indicating possible industrial emissions, and sulfate (SO_4_) concentrations exhibit a similar pattern (0.66–0.91 μg/m^3^), correlating with SO_2_ as it forms sulfate aerosols in the atmosphere.^53^ The SS concentrations range from 0.19 to 0.23 μg/m^3^, with a general increasing trend, with SS PM_2_._5_ showing corresponding trends, highlighting the contribution of SS to fine PM.^54^ The PM_2_._5_ concentrations, derived from MERRA-2 evaluation from various sources, range from 2.41E-08 to 2.93E-08 μg/m^3^, as the increasing trends show its adverse effects on human health and the environment.^49^

The trend summary of Figure 2 was further statistically assessed and presented in Table 1 for atmospheric particulate precursors over 45 years. The statistical indices showed that the mean, standard deviation (SD), standard error of the mean (SEM) and coefficient of variance (CV) showed low variability (CV values are all below 10%), suggesting consistent levels of pollutants and PM over the observed period. The skewness and kurtosis showed that OC and BC showed upward bell-shaped distribution, dust and dust PM_2_._5_ showed a similar downward bell-shaped distribution, SO_2_ and SO_4_ showed dissimilar upward segmented broad distribution, and SS and SS PM_2_._5_ showed a similar distribution. The negative skewness and negative kurtosis in most variables indicate that the distributions are left-skewed and flatter than the normal distribution. The consistent mean values and low SEM values suggest stable trends in the concentrations of these pollutants over time.

**Table 1:** Statistical analysis of atmospheric particulate precursors

	OC (kg/m^3^)	BC (kg/m^3^)	Dust (kg/m^3^)	Dust PM_2_._5_ (kg/m^3^)	SO_2_ (kg/m^3^)	SO_4_ (kg/m^3^)	SS (kg/m^3^)	SS - PM_2_._5_ (kg/m^3^)	PM _2_._5_ (kg/m^3^)
Min	1.52	0.19	79.1	17.80	0.50	0.66	18.50	2.92	24.10
Max	2.44	0.29	96.2	22.40	0.62	0.91	23.30	3.77	29.40
Sum	91.4	10.8	3910	898	25.70	36.6	930	151	1200
Mean	2.03	0.24	87	20	0.57	0.81	20.70	3.35	26.7
SEM	0.04	0.004	0.65	0.18	0.01	0.01	0.153	0.02	0.22
SD	0.24	0.03	4.36	1.21	0.04	0.06	1.02	0.15	1.48
Skewness	−0.41	−0.35	0.32	0.31	−0.50	−0.44	−0.27	−0.34	0.13
Kurtosis	−0.50	−0.51	−0.81	−0.92	−1.15	0.55	−0.01	1.50	−1.11
CV	11.77	10.82	5.01	6.06	6.56	6.50	4.95	4.52	5.54

**Figure 3:**
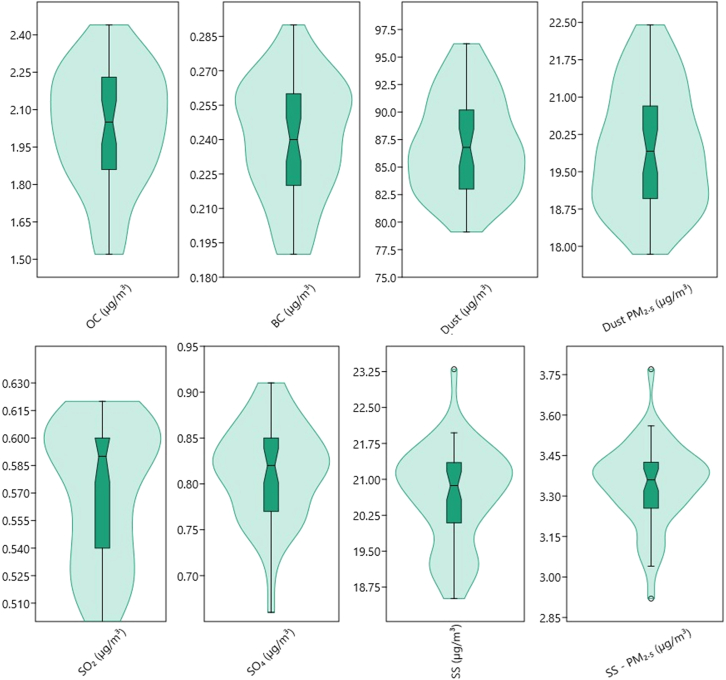
Violin boxplot of atmospheric particulate precursors

The violin boxplot (Figure 3) showed that BC and OC showed upward broad bell-like distribution thus attributed to normal symmetrically distributed around the median; whereas the dust and dust PM_2.5_ showed an opposite downwards bell-like distribution, respectively. SO_2_ and SO_4_ showed an upward bell-like distribution with the majority of the data near the median thus having shown fewer data at the upper levels than lower levels. SS and SS PM_2.5_ displayed broad with sparse narrow data ranges at the tails.

### Climate data

**Figure 4:**
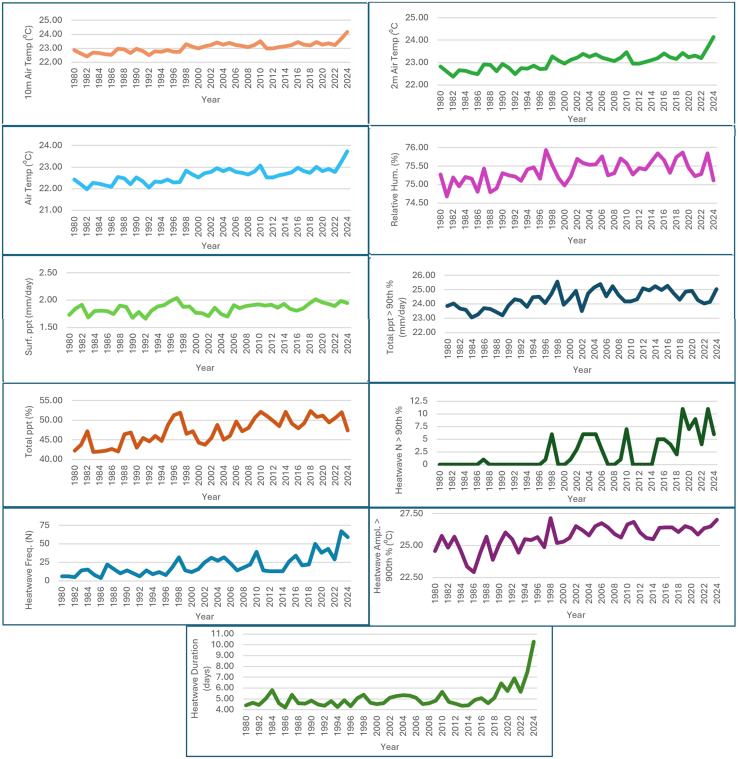
Annual trend chart of different climate conditions

The annual mean climate data of the Africa region spanning from 1980 to 2024 includes various climate metrics, such as air temperatures at different heights, surface humidity, precipitation, and heatwave statistics derived from MERRA-2 are presented in Figure 4 and Table S2. The temperature trends showed that 10 m air temperature (AT) showed a gradual show a gradual increase over the years. In 1980, the temperature was 22.86°C, rising to 24.17°C in 2024, indicating a warming trend, with 2 m AT and AT showed similar trends increasing from 22.83°C to 24.13°C, and 22.42°C–23.74°C in 1980–2024. The maximum temperature recording recorded rose from 26.75°C in 1980°C to 28.19°C in 2024 with the temperature trends suggesting a significant warming of the atmosphere over the 44 years is consistent with global climate change observations.^55^^,^^56^ The surface humidity remains relatively stable, fluctuating slightly around the 75% mark which suggests that the region’s relative humidity hasn’t been significantly affected by the warming temperature trend.^57^ The surface precipitation varied over the years but shows no clear upward or downward trend with 1980 (1.73 mm/day) and 2024 (1.94 mm/day). Total precipitation above the 90^th^ percentile ranges between 23.05 mm/day (1984) to 25.56 mm/day (1998), indicating extreme precipitation events, as the total precipitation as a percentage fluctuates but shows some years with higher percentages, indicating varying precipitation patterns.^58^ The heatwave (HW) statistics for climate data gave HW *N* > 90^th^ perc, which means the number of heatwave events above the 90^th^ percentile has shown a dramatic increase and decrease in the early years (1980s), it was consistently 0, but by 2024, it reached 6 that indicates more frequent extreme heat events. HW Freq, which is the frequency of heatwave events, also shows an increasing trend, as 1980 was 6, but by 2024 it increased to 59. The HW Ampl. >90^th^ perc, which is the amplitude of heatwaves above the 90^th^ percentile shows an increasing trend from 24.58°C in 1980°C to 27.02°C in 2024, indicating that heatwaves are becoming more intense.^59^^,^^60^ The HW Dur, which is the duration of heatwaves, increased from 4.41 days in 1980 to 10.27 days in 2024, suggesting that heatwaves last longer, as 2024 is predicted to have more heatwaves owing to the 6^th^ month’s trends from January to June 2024, which is in correlation with Engdaw et al.,^61^ studies in Africa.

The climate statistics presented in Table 2 provide a summary of the key climate metrics with temperature metrics indicating a warming trend over the 45 years, with a mean increase in temperatures and a slight right skewness indicating more frequent occurrences of higher temperatures. The humidity and precipitation metrics show relatively stable values with minimal skewness, indicating a consistent pattern over the years. The heatwave statistics highlight an increasing trend in both the frequency and intensity of heatwaves with the skewness and kurtosis values indicating that most years had moderate heatwave activity with occasional extreme events that significantly impact the average values. These climate statistics show a description of a warming climate with stable humidity and precipitation patterns but increasing heatwave events in both frequency and intensity.

**Table 2:** Statistical analysis of climate indices

Year	10m Air Temp (^°^C)	2 m Air Temp (^°^C)	Air Temp (^°^C)	Max Air Temp (^°^C)	Surf. Hum. (%)	Surf ppt (mm/day)	Total ppt >90th perc (mm/day)	Total ppt (%)	HW *N* > 90th perc	HW Freq (N)	HW Ampl. >90th perc (^°^C)	HW Dur. (days)
Min	22.42	22.37	21.98	26.20	74.69	1.66	23.05	41.95	0	4	22.93	4.22
Max	24.17	24.13	23.74	28.19	75.93	2.04	25.56	52.39	11	67	27.14	10.27
Sum	1037.65	1036.22	1017.68	1213.35	3391.29	83.40	1096.10	2130.05	105	944	1155.99	229.94
Mean	23.06	23.03	22.62	26.96	75.36	1.85	24.36	47.33	2.33	20.98	25.69	5.11
SEM	0.05	0.05	0.05	0.06	0.04	0.01	0.10	0.49	0.48	2.11	0.14	0.15
SD	0.35	0.35	0.34	0.37	0.30	0.09	0.64	3.26	3.25	14.18	0.93	1.04
Skewness	0.52	0.48	0.58	0.51	−0.12	−0.35	−0.09	−0.13	1.24	1.43	−1.00	3.27
Kurtosis	1.09	0.99	1.28	1.28	−0.39	−0.50	−0.81	−1.14	0.52	2.08	1.03	13.72
Coeff. var	1.50	1.51	1.51	1.39	0.40	5.06	2.62	6.89	139.17	67.62	3.62	20.33

**Figure 5:**
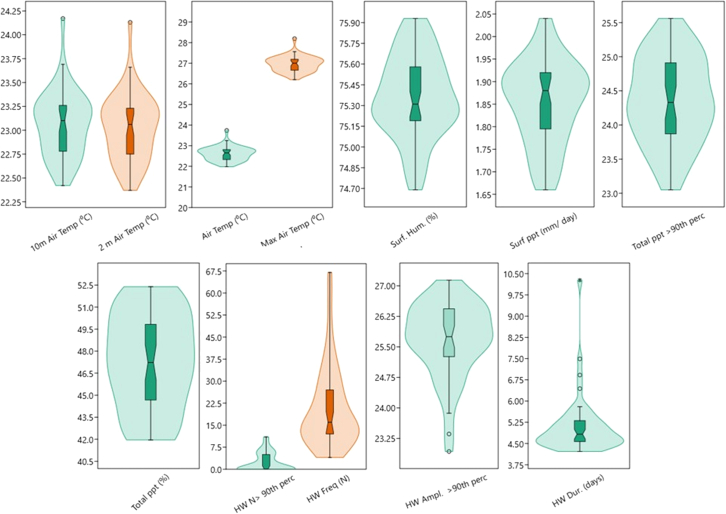
Violin boxplot of climate indices

Figure 5 shows the violin boxplot of climate indicators, as 10 m AT, 2 m AT, AT, and max AT showed similar broad, bell-like shaped indicates a normal distribution, with most data points concentrated around the median. Surf humidity showed a broad, bell-like shape indicates a normal distribution, with most data points around the median and lower distribution at base tails. Total precipitation had similar distribution while surface precipitation had wider distribution at the base tail than at the top tail. HW N showed the minimum temperature during heatwaves, whereas the HW frequency large data ranges with lower median levels. HW Ampl >95th percentile plot shows the amplitude of heatwaves above the 95th percentile, as the width at different points indicates the large ranges of data points in tandem with HW Dur (days).

### Particulate matter assessment

**Figure 6:**
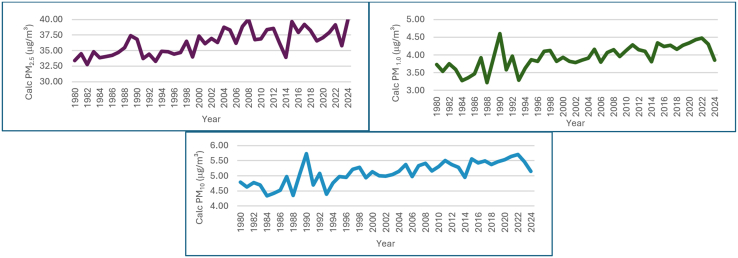
Annual trend chart of calculated PMs

The analysis of particulate matter (PM_2_._5_, PM_1_, and PM_10_ with a diameter of 2.5, 1.0 and 10 μg or less from 1980 to 2024, which is presented in Figure 6 and Table S3 with kg/m^3^ and converted to μg/m^3^ (1 kg/m^3^ = 10ˆ9 μg/m^3^). The trend over time showed that the calculated PM_2_._5_ (μg/m^3^) in 1980: 33.39 μg/m^3^ and 2024: 39.88 μg/m^3^, as the calculated PM_1_ (μg/m^3^) for 1980: 3.73 μg/m^3^ and 2024: 3.85 μg/m^3^ and the calculated PM_10_ (μg/m^3^) ranged from 1980: 4.79 μg/m^3^ to 2024: 5.14 μg/m^3^. The increasing trend in PM, especially PM_2_._5_, with significant peaks in early 2000s due to increase in industrial activities. These can penetrate deeply into the lungs, indicates worsening air quality and potential health impacts with prolonged exposure to higher concentrations of PM_2_._5_ is associated with respiratory and cardiovascular diseases.^49^ The slight increase in PM_1_ and PM_10_ also contributes to these health risks.^62^

The statistical analysis (Table 3) of PM data (1980–2024) showed key metrics with mean values of PM_2_._5_, PM_1_, and PM_10_ have remained relatively stable with a slightly increasing trend over the years. The standard deviations (SD) show the spread of the data around the mean, as the relatively low values indicate that the data points are close to the mean, implying consistent pollution levels over the years. The standard error of the mean (SEM) values was small, indicating precise mean estimates. The skewness values close to zero suggest a relatively symmetrical distribution of PM concentrations with negative skewness for PM_1_ (−0.31) and PM_10_ (−0.39) indicating a slight tendency for lower values, while the positive skewness for PM_2_._5_ (0.14) suggests a slight tendency for higher values. The negative kurtosis values indicate a flatter distribution compared to a normal distribution, implying fewer extreme values in the dataset. The coefficient of variation was relatively low, indicating low variability relative to the mean.

**Table 3:** Statistical PM data

Year	Calc PM _2_._5_ (μg/m^3^)	Calc PM_1_ (μg/m^3^)	Calc PM_10_ (μg/m^3^)
Min	32.7	3.22	4.34
Max	40	4.6	5.73
Sum	1630	177	229
Mean	36.3	3.94	5.1
SEM	0.30	0.049	0.056
SD	2.03	0.331	0.373
Skewness	0.14	−0.31	−0.39
Kurtosis	−1.10	−0.34	−0.58
Coeff. var	5.59	8.40	7.31

**Figure 7:**
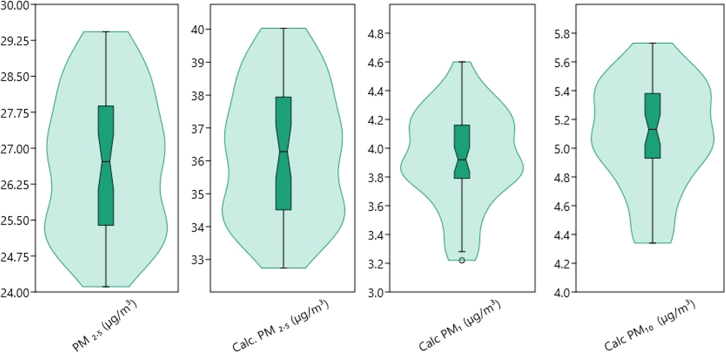
Violin boxplot of PM Indices

Figure 7 shows the violin boxplot of particulates, as PM_2.5_ and calculated PM_2.5_ displays similar broad, bell-like shape indicating a normal distribution, with most data centered around the median. Calculated PM_1.0_ and PM_10.0_ showed similar dissimilar distribution of upper tail higher than lower tail.

### Climate modeling assessment

The climate modeling data analysis was conducted for a five-year gradient (Table 4) as the heat frequency (HF) showed a significant increasing trend from 9.20 in 1980–1984 to 59.00 in 2024∼, as increased HF indicates more frequent occurrences of extreme heat day, which can lead to higher instances of heat-related health issues, increased energy demand for cooling, and exacerbated drought conditions.^63^^,^^64^ The heat index (HI) remained relatively stable with a gradual increase from 28.36 to 31.74 with a higher HI suggesting that the combination of temperature and humidity is making it feel hotter, which can have severe implications on human health, agriculture, and energy consumption.^65^ Heatwave index (HWI) and heatwave severity index (HWSI) both show a decreasing trend, with HWI declining from −12.28 to −30.54, and HWSI from −169.87 to −47.08 with the decrease suggesting that while the frequency of heatwaves is increasing, the severity of individual heatwaves is decreasing, as frequent heatwaves can still strain public health systems and infrastructure.^66^ The precipitation concentration index (PCI) values fluctuate slightly but generally remain high, indicating a concentration of rainfall in fewer events; with high PCI values suggesting that precipitation is occurring in more intense bursts, leading to increased risks of flooding and soil erosion, which can negatively impact agriculture and infrastructure.^67^ The standardized precipitation index (SPI) shows a decreasing trend from −17.15 to −28.07, as a negative SPI suggests worsening drought conditions, which can impact water resources, agriculture, and food security.^68^ The climate extreme index (CEI) increases from −49.95 to −38.86, as higher CEI values indicate more frequent and severe climate extremes, which can lead to increased risks of natural disasters, challenges to human health, and significant economic impacts.^69^ The hydro-climate index (HCI) shows a slight increasing trend from 7.89 to 8.35, as higher HCI values indicate more extreme hydro-climate conditions, such as intense rainfall events leading to floods and prolonged dry periods causing droughts as this variability poses challenges to water resource management and agricultural planning.^70^

**Table 4:** Climate modeling data analysis for Africa

Five-year gradient	HF	HI	HWI	HWSI	PCI	SPI	CEI	HCI
1980–1984	9.20	28.36	−12.28	−169.87	197.03	−17.15	−49.95	7.89
1985–1989	12.00	28.47	−12.66	−141.78	195.44	−17.77	−50.07	7.83
1990–1994	10.60	28.58	−12.61	−126.74	201.22	−17.15	−49.83	8.06
1995–1999	16.80	28.94	−15.35	−93.68	204.62	−16.52	−49.73	8.19
2000–2004	22.20	29.68	−18.46	−66.93	204.49	−27.38	−70.33	8.19
2005–2009	21.80	29.62	−17.07	−64.15	206.67	−26.34	−70.07	8.27
2010–2014	18.40	29.29	−15.79	−81.48	206.39	−27.57	−70.08	8.26
2015–2019	30.60	29.69	−22.27	−49.75	206.95	−26.78	−70.06	8.29
2020–2024	47.20	30.14	−27.84	−42.89	204.01	−28.19	−39.12	8.17
2024∼	59.00	31.74	−30.54	−47.08	208.65	−28.07	−38.86	8.35

### Spearman and Pearson correlation

The Spearman and Pearson correlation was analyzed and presented in Table 5 to understand the relationships between different particulate and climatic variables. Spearman’s correlation assesses the strength and direction of the association between two ranked variables, while Pearson’s correlation evaluates the linear relationship between two continuous variables. Spearman’s correlation matrix showed that OC and BC had a very high correlation (0.97), indicating a strong association between organic and black carbon is robust across both rank and linear metrics. Dust and dust PM_2_._5_ showed a near-perfect correlation (0.99), suggesting that the presence of dust is strongly associated with PM_2_._5_ levels. OC and Calc PM_1_ and PM_10_ had a high correlation (0.96; 0.92), showing a strong relationship between OC and calculated PM levels. SO_2_ and SO_4_ had a moderate correlation (0.48), indicating some degree of association between sulfur dioxide and sulfate.^71^ SS and SO_2_ had a high correlation (0.72), suggesting that SS is significantly associated with sulfur dioxide. 10m AT, 2m AT, and AT had a perfect correlation (1.00), indicating these temperature measurements are identical in rank-based perspectives. Heatwave variables (number, frequency, magnitude, amplitude, and duration) had significant correlations among themselves, particularly between heatwave number >90th percentile and heatwave frequency (0.91), also 10 m AT, 2 m AT, and AT correlate strongly with Heatwave (Number, Frequency, Amplitude). Pearson’s Correlation Matrix showed that OC and BC (0.94) had a very strong correlation linear relationship, similar to Spearman’s, indicating a strong linear relationship with dust and dust PM_2_._5_, emphasizing the association seen in Spearman’s. OC/BC and Calc PM_1_ and PM_10_ had a similar high correlation, confirming a strong linear relationship.^64^ SO_2_ and SO_4_ had a moderate correlation (0.36), suggesting a weaker linear association compared to Spearman’s. SS and SO_2_ had a high correlation (0.80), consistent with Spearman’s results.^72^ 10m AT, 2m AT, and AT had a perfect correlation (1.00), indicating perfect linear relationships. Heatwave variables: strong correlations among themselves, with notable high values for heatwave number >90^th^ and frequency (0.92). SO_2_ and PM_2_._5_/Calc. PM_2_._5_/Calc PM_1_/Calc PM_10_/10m AT/2m AT/AT had a strong correlation (0.73–0.79) SS and PM_2_._5_ showed a similarly strong correlation with PM_2_._5_/Calc PM_2_._5_/10m AT/2m AT/AT implying great interaction.

**Table 5:** Spearman’s and Pearson’s correlation analysis

Spearman’s Correlation																									
Pearson΄s Correlation		OC	BC	Dust	Dust PM_2_._5_	SO_2_	SO_4_	SS	SS - PM_2_._5_	PM _2_._5_	Calc PM _2_._5_	Calc PM_1_	Calc PM_10_	10m AT	2 m AT	AT	Surf. Hum.	Surf ppt	Total ppt >90th	Total ppt	HW *N* > 90th	HW Freq (N)	HW Magn >90th	HW Ampl	HW Dur
	OC		**0.97**	0.35	*0.40*	*0.69*	0.25	*0.51*	*0.55*	*0.56*	*0.56*	**0.96**	**0.92**	*0.53*	*0.52*	*0.53*	*0.51*	0.29	*0.44*	*0.54*	*0.52*	*0.52*	−0.21	*0.42*	0.37
	BC	**0.94**		*0.41*	*0.46*	**0.72**	0.29	*0.53*	*0.56*	*0.61*	*0.61*	**0.95**	**0.93**	*0.54*	*0.54*	*0.55*	*0.49*	0.29	*0.45*	*0.56*	*0.48*	*0.50*	−0.22	*0.45*	0.34
	Dust	0.37	*0.43*		**0.99**	*0.62*	*0.59*	*0.58*	*0.59*	**0.95**	**0.95**	*0.51*	*0.61*	*0.50*	*0.51*	*0.50*	0.15	−0.02	*0.42*	*0.20*	0.34	*0.45*	−0.12	*0.58*	0.24
	Dust PM_2_._5_	0.39	*0.46*	**1.00**		*0.67*	*0.60*	*0.63*	*0.64*	**0.97**	**0.97**	*0.56*	*0.66*	*0.55*	*0.55*	*0.55*	0.20	0.04	*0.45*	*0.26*	*0.40*	*0.50*	−0.16	*0.60*	0.29
	SO_2_	*0.67*	*0.57*	0.65	*0.67*		*0.48*	**0.81**	**0.81**	**0.75**	**0.75**	**0.75**	**0.81**	**0.81**	**0.81**	**0.80**	*0.55*	0.34	*0.61*	*0.61*	*0.66*	**0.72**	−0.26	**0.76**	*0.44*
	SO_4_	0.19	0.23	0.52	*0.54*	0.36		*0.40*	*0.46*	*0.60*	*0.59*	*0.48*	*0.54*	0.35	0.36	0.35	0.25	−0.04	0.15	0.14	0.32	0.31	−0.15	0.37	0.23
	SS	*0.50*	*0.52*	0.63	*0.66*	**0.80**	0.37		**0.93**	**0.70**	**0.70**	*0.59*	*0.65*	**0.84**	**0.84**	**0.83**	*0.43*	0.35	*0.64*	*0.51*	*0.62*	**0.72**	−0.31	**0.75**	*0.44*
	SS - PM_2_._5_	0.41	*0.41*	0.58	*0.62*	*0.65*	*0.42*	**0.93**		**0.73**	**0.73**	*0.63*	*0.67*	**0.85**	**0.85**	**0.86**	*0.44*	0.29	*0.53*	*0.43*	**0.71**	**0.79**	−0.38	**0.71**	*0.53*
	PM _2_._5_	*0.55*	*0.61*	0.96	**0.98**	**0.76**	*0.57*	**0.74**	**0.70**		**1.00**	**0.70**	**0.78**	*0.62*	*0.62*	*0.62*	0.30	0.12	*0.51*	0.35	*0.47*	*0.57*	−0.20	*0.63*	0.34
	Calc PM _2_._5_	*0.55*	*0.61*	0.97	**0.98**	**0.76**	*0.56*	**0.75**	**0.71**	**1.00**		**0.70**	**0.78**	*0.62*	*0.62*	*0.62*	0.30	0.12	*0.51*	0.35	*0.47*	*0.57*	−0.20	*0.63*	0.34
	Calc PM_1_	**0.96**	**0.94**	0.48	*0.51*	**0.73**	*0.43*	*0.56*	*0.48*	*0.66*	*0.66*		**0.98**	*0.59*	*0.58*	*0.59*	*0.52*	0.26	*0.47*	*0.55*	*0.55*	*0.57*	−0.27	*0.48*	*0.40*
	Calc PM_10_	**0.94**	**0.94**	0.58	*0.61*	**0.79**	*0.46*	*0.64*	*0.55*	**0.75**	**0.75**	**0.99**		*0.64*	*0.64*	*0.64*	*0.52*	0.25	*0.52*	*0.55*	*0.58*	*0.60*	−0.25	*0.56*	*0.40*
	10m AT	*0.49*	*0.51*	0.51	*0.55*	**0.76**	0.25	**0.87**	**0.85**	*0.64*	*0.64*	*0.51*	*0.58*		**1.00**	**1.00**	*0.61*	0.28	*0.59*	*0.50*	**0.85**	**0.90**	−0.27	**0.77**	*0.65*
	2 m AT	*0.49*	*0.51*	0.51	*0.55*	**0.77**	0.25	**0.87**	**0.84**	*0.64*	*0.64*	*0.51*	*0.58*	**1.00**		**1.00**	*0.60*	0.27	*0.59*	*0.49*	**0.85**	**0.89**	−0.26	**0.77**	*0.64*
	AT	*0.48*	*0.51*	0.51	*0.55*	**0.76**	0.25	**0.87**	**0.85**	*0.64*	*0.64*	*0.51*	*0.57*	**1.00**	**1.00**		*0.61*	0.28	*0.58*	*0.49*	**0.86**	**0.90**	−0.28	**0.75**	*0.66*
	Surf. Hum	*0.52*	*0.49*	0.10	0.14	*0.61*	0.17	*0.40*	0.35	0.25	0.25	*0.51*	*0.51*	*0.49*	*0.50*	*0.48*		0.29	0.50	*0.56*	*0.61*	*0.58*	−0.13	*0.46*	*0.42*
	Surf ppt	0.21	0.21	−0.01	0.04	0.31	0.07	*0.40*	0.36	0.11	0.11	0.21	0.23	0.28	0.28	0.28	0.31		0.19	**0.84**	0.28	0.28	−0.30	0.26	0.23
	Total ppt >90th	*0.51*	*0.50*	0.42	*0.43*	*0.67*	0.12	*0.62*	*0.47*	*0.50*	*0.50*	*0.50*	*0.55*	*0.56*	*0.57*	*0.55*	*0.48*	0.21		0.39	*0.41*	0.39	0.02	*0.55*	0.09
	Total ppt	*0.55*	*0.56*	0.22	0.26	*0.64*	0.14	*0.53*	*0.38*	0.36	0.36	*0.54*	*0.57*	*0.47*	*0.47*	*0.45*	*0.57*	**0.80**	*0.41*		*0.42*	*0.43*	−0.31	*0.48*	0.24
	HW *N* > 90th	*0.48*	*0.47*	0.26	0.31	*0.62*	0.30	*0.59*	*0.62*	*0.42*	*0.42*	*0.52*	*0.53*	**0.76**	**0.75**	**0.76**	*0.52*	0.30	0.39	*0.42*		**0.91**	−0.31	*0.65*	**0.79**
	HW Freq (N)	*0.48*	*0.47*	0.31	0.36	*0.65*	0.20	**0.71**	**0.75**	*0.47*	*0.47*	*0.48*	*0.52*	**0.87**	**0.87**	**0.88**	*0.49*	0.36	0.35	*0.44*	**0.92**		*−0.43*	*0.69*	**0.86**
	HW Magn >90th	−0.23	−0.23	−0.15	−0.17	−0.28	−0.15	−0.31	−0.36	−0.23	−0.22	−0.25	−0.26	−0.30	−0.30	−0.30	−0.19	−0.35	−0.05	−0.38	−0.39	*−0.45*		−0.18	−0.37
	HW Ampl >90th	*0.51*	*0.52*	*0.52*	*0.52*	**0.76**	0.21	**0.70**	*0.54*	*0.58*	*0.59*	*0.52*	*0.58*	**0.72**	**0.72**	**0.71**	*0.45*	0.25	0.62	*0.53*	*0.55*	*0.59*	−0.22		*0.49*
	HW Dur	0.27	0.27	0.24	0.28	0.37	−0.01	*0.54*	*0.61*	0.34	0.34	0.24	0.27	**0.72**	**0.71**	**0.73**	0.19	0.30	0.15	0.21	*0.67*	**0.85**	−0.31	0.39	

Note: Statistical significance is indicated by a *p* value ≤0.05. Strong correlation: Bold (0.70–1.00), medium correlation: italics (0.40–0.69), and weak correlation: (0.0–0.39).

### Scattered plot

**Figure 8:**
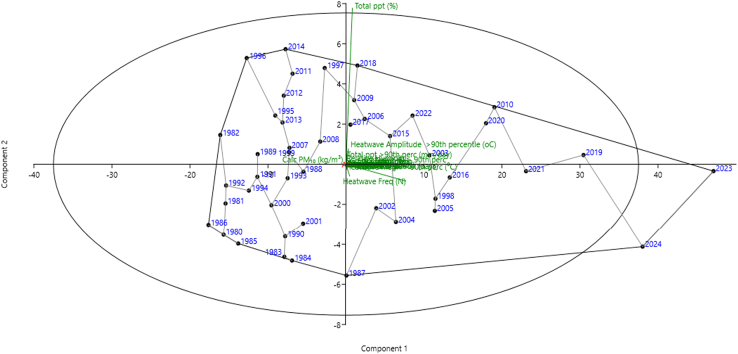
Scatterplot with spanning linked trees at *p* ≤ 0.05

A scatterplot is used to visualize the relationship between two continuous variables from factor analysis (principal component analysis, PCA) by plotting each variable on one axis against another axis to understand patterns, correlations, and potential outliers. Figure 8 shows the scatterplot conducted for all climatic factors and particulates for Africa, as the linkages were from 2014 to 2018, 2010, 2023 to 2024, 1987, 1984, 1983, 1985, 1980, 1986, and 1982 to 1996 with interlinkages and corresponding correlations points, as positive correlation trends point upward from left to right and vice versa downward (negative correlation), while no correlation was scattered without a clear pattern as shown in Total ppt (%) and also annual years of 2023 and 2024, thus signifies the strength and direction of a relationship at significance *p* value ≤0.05.

### Dendrogram clustering

**Figure 9:**
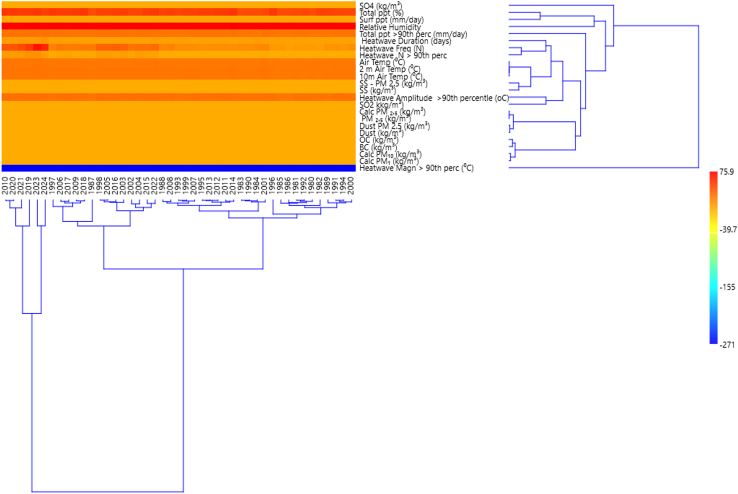
Dendrogram clustering with heatmap

Dendrogram clustering is used to visualize hierarchical relationships among variables or observations, which represents how variables (or data points) are grouped based on their similarity. Figure 9 shows the dendrogram clustering with annual trends used to visualize cluster groups via the similarity index (0.993–1.000) and parameter clusters from the horizontal view. The parameter clusters showed several tree linkages with Calc. PM_2_._5_ and PM_2_._5_ linked to dust PM_2_._5_ and dust that is further linked to OC/BC and Calc PM_10_ and Calc. PM 1. The linkages continue via an increasing spanning tree with other climate parameters and links to SO_4_ and finally linked to heatwave magnitude >90^th^ percentile. The vertical clusters showed three segments of clusters with adjoining clusters; the first clusters (2010 and 2020 linked to 2021 and 2019 joined to 2023/2024); the second clusters (1997 linked to 2006/2007/2009/2018 and 1987 that links again with other cluster groups of 1998/2005/2016/2003 and 2002/2004/2015/2022) and the third clusters (group 1a of 1988/2008/1993/1999/2007/1995/2013/2012/2011/2014 linked to group 1b 1983/19990/1984/2001 and group 2a of 1996 linked to group 2b 1981/1992/1980/1985/1986 and linked to group 2c of 1989/1991/1994/2000). The second and third clusters link at 0.9985, which is further connected to the first cluster with a similarity index of 0.993. The color bar represents the similarity index, which indicates closeness to variation.^73^ The data matrices were dimensionally reduced to help identify the main factors influencing clustering suggesting similar sources or influences or vice versa.^74^

## Discussion

**Figure 10:**
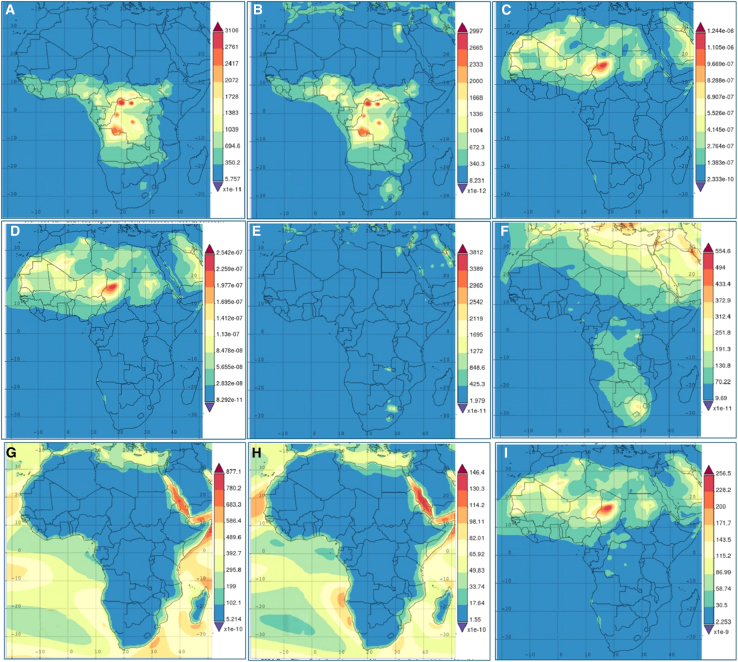
Average surface mass concentration of atmospheric components over Africa (kg/m^3^) (A) OC (organic carbon); (B) BC (black carbon); (C) dust; (D) dust PM_2_._5_; (E) SO_2_ (sulfur dioxide); (F) SO_4_ (sulfate); (G) SS (sea salt); (H) SS - PM_2_._5_; (I) PM2.5 (particulate matter 2.5).

This research delves into the atmospheric PM and climatic changes across Africa, using data from MERRA-2 to assess trends and implications for the continent. The study’s findings (Figure 10) reveal a significant increase in aerosol concentrations, specifically OC, black carbon (BC), sulfur oxide (SO_2_ and SO_4_), and particulate matter (PM_2_._5_) with increasing surface atmospheric mass concentration due to intensified anthropogenic activities, such as industrialization, urbanization, and the surge in vehicle emissions.^75^^,^^76^^,^^77^ The rising levels of PM_2_._5_ are alarming due to their direct association with respiratory and cardiovascular diseases. The data suggest a growing public health risk as this fine PM can penetrate deep into the lungs, leading to severe health issues, particularly in densely populated urban areas.

According to WHO air quality guidelines,^78^^,^^79^ exposure to PM_2_._5_ can cause diseases such as stroke, lung cancer, and chronic obstructive pulmonary disease (COPD), as new research shows PM also impacts psychological and behavioral problems including anxiety, depression, and attention deficit hyperactivity disorder (ADHD).^79^ Moreover, high dust concentrations, especially in arid and semi-arid regions of Africa, exacerbate the situation by contributing to soil erosion, reduced visibility, and additional respiratory problems.^72^^,^^80^ The climatic impacts of aerosols are profound, influencing the Earth’s radiation balance in various ways, as OC tends to scatter sunlight, thereby cooling the atmosphere, while BC absorbs sunlight, leading to warming.^34^ This dual effect creates a complex interaction that can alter temperature and precipitation patterns across the continent with dust and sulfate aerosols playing a significant role in cloud formation and can modulate precipitation, influencing both the frequency and intensity of rain events.^81^^,^^82^^,^^83^ Such changes are crucial as they can lead to more extreme weather conditions, including prolonged droughts or intense rainfall, both of which have far-reaching effects on agriculture, water resources, and overall ecosystem stability.^84^^,^^85^

**Figure 11:**
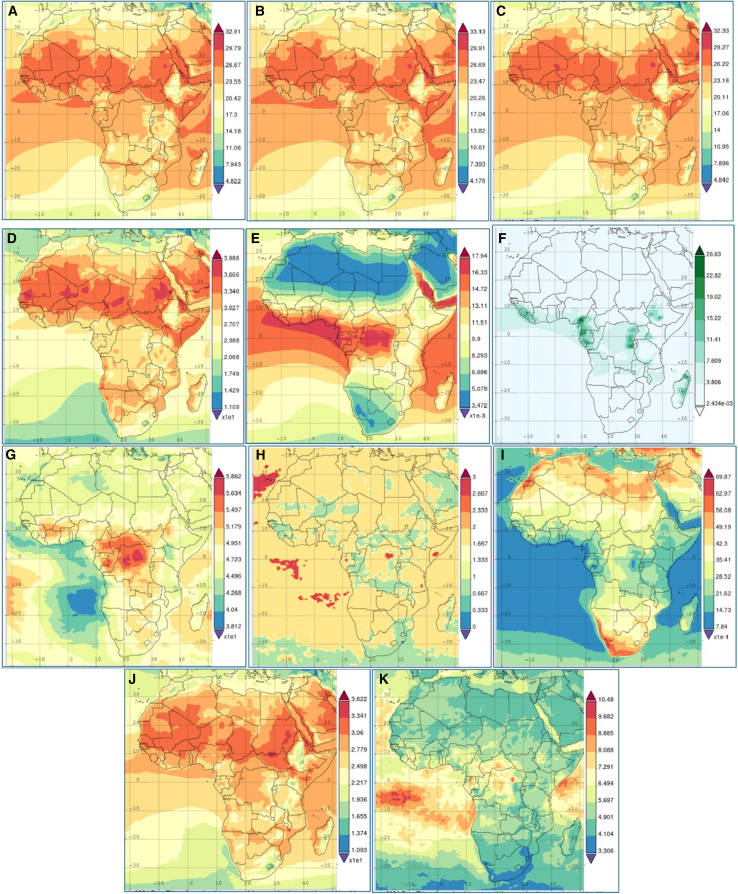
Average climatic characteristics data over Africa (A) 10 m air temperature (°C); (B) 2 m air temperature (°C); (C) air temperature (°C) amplitude > temperature (°C); (E) surface humidity (%); (F) surf precipitation (mm/day); (G) total precipitation (%); (H) HW (heatwave) frequency (N); (I) HW magnitude (K); (J) HW amplitude >90th percentile (°C); (K) HW duration (N).

The consistent rise in air temperatures across all measured altitudes (ground level AT, 2 m AT, and 10 m AT), indicates a clear warming trend in Africa (Figure 11), aligning with global climate change observations, as this warming is not just a statistical anomaly but a critical indicator of the shifting climate dynamics in the region.^86^ The increased frequency and intensity of heat waves pose significant risks to human health, particularly in vulnerable populations, such as children, the elderly, and those with pre-existing health conditions.^87^ Heatwaves also have a cascading effect on agriculture, reducing crop yields and increasing water demand in an already water-stressed region.^88^ The need for adaptive strategies, such as improved irrigation systems and heat-resistant crop varieties, is more urgent than ever, as the study also highlights the changing precipitation patterns across Africa, with an increase in extreme weather events. While overall average precipitation may not show a drastic shift, the distribution and intensity of rainfall events have changed. This variability can lead to severe flooding in some areas and prolonged droughts in others, as significant environmental changes threaten water security, as inconsistent rainfall can deplete water resources, impact hydroelectric power generation, and increase competition for water among agricultural, industrial, and domestic users. The rising trend in total precipitation above the 90th percentile suggests a shift toward more extreme rainfall events, which could lead to flash floods, soil erosion, and infrastructure damage.^88^

The climate modeling data (Table 4 and Figure 11) for Africa between 1980 and 2024 revealed a significant shift in HF, HI, heatwave indices, and precipitation patterns, which have profound regional implications due to the continent’s diverse climates and geographies.^89^ In Northern Africa, increasing HF and heat indices exacerbate risks related to heat waves and heat-related health issues with the region’s arid nature, coupled with increased precipitation variability, threatening water security, and exacerbating drought conditions.^82^ These changes necessitate the need for improved water management strategies, particularly for regions dependent on river systems like the Nile.

In Sub-Saharan Africa, the implications vary widely across different sub-regions with West Africa facing more frequent heatwaves, impacting human health, labor productivity, and energy demand. Recent reports by Aljazerra^90^ have highlighted significant heatwave events in the Sahel and West Africa regions. In April 2024, an intense heatwave with temperatures exceeding 45°C was recorded in Mali and Burkina Faso, as these extreme heat events, attributed to human-caused climate change, resulted in numerous heat-related deaths and hospitalizations.^91^

The validation of the HWI against such observed data underscores its effectiveness in capturing the severity and impact of heatwaves in the region. Rainfall variability in this region threatens agricultural productivity, heightening food security risks, and increasing the likelihood of urban flooding due to inadequate drainage systems.^49^ East Africa experiences similar challenges, with higher heat indices and more frequent heatwaves affecting both human health and agricultural productivity via the region’s precipitation variability disrupts growing seasons in Somalia, Kenya, Sudan, South Sudan, Ethiopia, and Eritrea.^92^^,^^93^ The study by PIK, BMZ, and GIZ^93^ and Kilimo Kwanza^94^ on climate risk and drought profile of East Africa shows that extreme long dry and short wet period complicates water resource management thus increasing temperature by 1.7–3.5°C and increasing heatwave projection from 1.6% in 2000 to 10.4% in 2080. These empirical findings indicate a dual threat of drought and flooding, which complicates water availability and heightens risks of landslides, climate change, and soil erosion.^95^^,^^96^

Southern Africa is particularly vulnerable to droughts, with significant increases in HF^97^ and negative trends in the SPI, indicating worsening drought conditions, which is in tandem with climate change writers^98^ studies in Johannesburg, South Africa. These trends threaten water supplies, agricultural outputs, and hydropower generation, which could destabilize local social economies and food security.^99^^,^^100^ In Central Africa, the equatorial climate and high rainfall make the region prone to both excessive flooding and periodic droughts with changes in temperature and precipitation disrupting ecosystems, particularly in the Congo Basin, threatening biodiversity, forest dynamics, and carbon sequestration (1.1 billion ton carbon loss per year), which contributes to global climate change.^72^^,^^101^^,^^102^^,^^103^^,^^104^ These regional impacts underscore the need for tailored adaptation strategies across Africa to address the diverse and evolving challenges posed by climate change. Effective measures include investing in resilient infrastructure, enhancing water management, improving agricultural practices, and protecting biodiversity, all of which are crucial for mitigating the adverse effects of climate change and safeguarding the continent’s socio-economic stability.

### Study limitation and recommendation

Potential errors from satellite-based aerosol retrieval methods, such as those used in MERRA-2, can introduce inaccuracies due to various factors. These include surface characterization issues, aerosol proximity to clouds, and challenges in interpreting the results. For instance, retrievals near clouds can be contaminated by cloud-scattered light, leading to substantial errors. Additionally, the algorithms used to infer aerosol properties may have limitations in distinguishing between different aerosol types, especially in regions with complex mixtures of aerosols. Missing or incomplete data can significantly impact the reliability of trend analysis. In the context of this study, gaps in the data could arise from sensor malfunctions, data transmission errors, or periods when satellite observations are unavailable due to cloud cover or other obstructions. These gaps can lead to biased estimates and reduce the statistical power of the analysis. To mitigate these issues, we employed data imputation techniques and cross-validated our findings with multiple research studies and physical news to ensure robustness and accuracy.

To mitigate emissions, it is recommended to implement vehicle emission standards similar to Euro 6 regulations in major African cities as recommended by UNEP^105^ and Environmental Compliance Institute.^106^ This would significantly reduce the levels of PM_2_._5_ and other harmful pollutants. Investing in advanced irrigation systems and water conservation techniques is crucial to address the variability in precipitation and ensure water security, which includes rainwater harvesting and efficient water usage practices. Promoting the use of heat-resistant crop varieties and sustainable farming practices will enhance agricultural productivity and resilience against climate extremes. Implementing conservation strategies to protect biodiversity, particularly in vulnerable ecosystems like the Congo Basin, is essential. This includes reforestation projects and the establishment of protected areas. Addressing the rising levels of PM_2_._5_ by improving air quality monitoring and implementing public health initiatives is vital. Referencing WHO air quality guidelines to contextualize findings and develop targeted interventions will help in strengthening public health measures. These tailored adaptation strategies are crucial for mitigating the adverse effects of climate change and safeguarding the continent’s socio-economic stability.

### Conclusion

The data collected over the past 44 years presents compelling evidence of climate change across Africa, marked by rising air temperatures, more frequent and intense heat waves, and shifting precipitation patterns. These changes underscore the urgency for adaptive measures to mitigate the growing impacts on human health, agriculture, water resources, and ecosystems. The rising trend in PM concentrations further emphasizes the need for continued monitoring and robust pollution control strategies to protect public health and the environment. Strong correlations between key environmental variables (AT, 2 m AT, 10 m AT, HW frequency, HW number, SO2, PM_1.0_, PM_2.5_, PM_10_, SS, and dust) highlight the complex interplay between climate factors, necessitating a comprehensive approach to climate adaptation and mitigation. The regional analyses reveal that climate change impacts vary significantly across Africa, reflecting the continent’s diverse climates and geographies. Northern Africa faces exacerbated risks from heatwaves and water scarcity, while Sub-Saharan Africa contends with increased heat, precipitation variability, and heightened risks to agriculture and urban infrastructure and also ecosystem change in southern Africa, as well as human health impact, increasing environmental and energy demand to scare water resources. So, addressing these challenges requires tailored adaptation strategies, regional cooperation, and international support. By investing in resilient infrastructure, enhancing water management, improving agricultural practices, and developing comprehensive health response plans, Africa can build resilience against evolving climate conditions and secure a sustainable future for its diverse populations and landscapes.

## Resource availability

### Lead contact

Further information and requests should be directed to and will be fulfilled by the lead contact, Daniel O. Omokpariola (omeodisemi@gmail.com).

### Materials availability

This study did not generate unique reagents.

### Data and code availability

• The source data and maps can be accessed from NASA’s GES DISC https://disc.gsfc.nasa.gov, these data were produced by the MERRA-2 projects with NASA.
• No original codes were used in the processing or plotting of data in this article. All statistical visualization and data processing were done using PAST v4 software and Microsoft Excel.
• Any additional information required to reanalyze the data reported in this article is available from the lead contact upon request.

## Acknowledgments

Our acknowledgment goes to MERRA-2 scientists and associated NASA personnel for producing the data used in this research effort in addition to the analysis and visualization used from GIOVANNI online data system, developed, and maintained by NASA GES DISC.

## Author contributions

D.O.O.: writing – conceptualization, methodology, investigation, validation, visualization, original draft and writing – review and editing.

## Declaration of interests

The author declares no competing interests.

## STAR★Methods

### Key resources table

**Table:** 

REAGENT or RESOURCE	SOURCE	IDENTIFIER
**Chemicals**		

Organic carbon (kg/m^3^)	M2TMNXAER_5_12_4_OCSMASS^107^	OC
Black carbon (kg/m^3^)	M2TMNXAER_5_12_4_BCSMASS^107^	BC
Dust (kg/m^3^)	M2TMNXAER_5_12_4_DUSMASS^107^	Dust
Dust–PM_2_._5_ (kg/m^3^)	M2TMNXAER_5_12_4_DUSMASS25^107^	Dust–PM_2_._5_
Sea Salt–PM_2_._5_ (kg/m^3^)	M2TMNXAER_5_12_4_SSSMASS25^107^	Sea Salt–PM_2_._5_
Sea Salt (kg/m^3^)	M2TMNXAER_5_12_4_SSSMASS^107^	Sea Salt
Sulfate (kg/m^3^)	M2TMNXAER_5_12_4_SO4SMASS^107^	SO_4_
Sulfur dioxide (kg/m^3^)	M2TMNXAER_5_12_4_SO4SMASS^107^	SO_2_
Air density (kg/m^3^)	M2TMNXFLX_5_12_4_RHOA^107^	Airdens
Dust (DOO1) due to Wet deposition (kg/m^2^/s)	M2TMNXADG_5_12_4_DUWT001^107^	DUW001
Dust (DOO2) due to Wet deposition (kg/m^2^/s)	M2TMNXADG_5_12_4_DUWT002^107^	DUW002
Dust (DOO3) due to Wet deposition (kg/m^2^/s)	M2TMNXADG_5_12_4_DUWT003^107^	DUW003
Dust (DOO4) due to Wet deposition (kg/m^2^/s)	M2TMNXADG_5_12_4_DUWT004^107^	DUW004
Dust (DOO1) due to Dry deposition (kg/m^2^/s)	M2TMNXADG_5_12_4_DUDP001^107^	DUDP001
Dust (DOO2) due to Dry deposition (kg/m^2^/s)	M2TMNXADG_5_12_4_DUDP002^107^	DUDP002
Dust (DOO3) due to Dry deposition (kg/m^2^/s)	M2TMNXADG_5_12_4_DUDP003^107^	DUDP003
Dust (DOO4) due to Dry deposition (kg/m^2^/s)	M2TMNXADG_5_12_4_DUDP004^107^	DUDP004
Dust (DOO1) due to Convective Scavenging deposition (kg/m^2^/s)	M2TMNXADG_5_12_4_DUSV001^107^	DUSV001
Dust (DOO2) due to Convective Scavenging deposition (kg/m^2^/s)	M2TMNXADG_5_12_4_DUSV002^107^	DUSV002
Dust (DOO3) due to Convective Scavenging deposition (kg/m^2^/s)	M2TMNXADG_5_12_4_DUSV003^107^	DUSV003
Dust (DOO4) due to Convective Scavenging deposition (kg/m^2^/s)	M2TMNXADG_5_12_4_DUSV004^107^	DUSV004
Dust (DOO1) due to Emission deposition (kg/m^2^/s)	M2TMNXADG_5_12_4_DUEM001^107^	DUEM001
Dust (DOO2) due to Emission deposition (kg/m^2^/s)	M2TMNXADG_5_12_4_DUEM002^107^	DUEM002
Dust (DOO3) due to Emission deposition (kg/m^2^/s)	M2TMNXADG_5_12_4_DUEM003^107^	DUEM003
Dust (DOO4) due to Emission deposition (kg/m^2^/s)	M2TMNXADG_5_12_4_DUEM004^107^	DUEM004
Dust (DOO1) due to Sedimentation deposition (kg/m^2^/s)	M2TMNXADG_5_12_4_DUSD001^107^	DUSD001
Dust (DOO2) due to Sedimentation deposition (kg/m^2^/s)	M2TMNXADG_5_12_4_DUSD002^107^	DUSD002
Dust (DOO3) due to Sedimentation deposition (kg/m^2^/s)	M2TMNXADG_5_12_4_DUSD003^107^	DUSD003
Dust (DOO4) due to Sedimentation deposition (kg/m^2^/s)	M2TMNXADG_5_12_4_DUSD004^107^	DUSD004
Sea salt (SS001) due to Dry (kg/m^2^/s)	M2TMNXADG_5_12_4_SSDP001^107^	SSDP001
Sea salt (SS002) due to Dry (kg/m^2^/s)	M2TMNXADG_5_12_4_SSDP002^107^	SSDP002
Sea salt (SS003) due to Dry (kg/m^2^/s)	M2TMNXADG_5_12_4_SSDP003^107^	SSDP003
Sea salt (SS004) due to Dry (kg/m^2^/s)	M2TMNXADG_5_12_4_SSDP004^107^	SSDP004
Sea salt (SS001) due to Wet (kg/m^2^/s)	M2TMNXADG_5_12_4_SSWT001^107^	SSWT001
Sea salt (SS002) due to Wet (kg/m^2^/s)	M2TMNXADG_5_12_4_SSWT002^107^	SSWT002
Sea salt (SS003) due to Wet (kg/m^2^/s)	M2TMNXADG_5_12_4_SSWT003^107^	SSWT003
Sea salt (SS004) due to Wet (kg/m^2^/s)	M2TMNXADG_5_12_4_SSWT004^107^	SSWT004
Sea salt (SS001) due to Convective Scavenging (kg/m^2^/s)	M2TMNXADG_5_12_4_SSSV001^107^	SSSV001
Sea salt (SS002) due to Convective Scavenging (kg/m^2^/s)	M2TMNXADG_5_12_4_SSSV002^107^	SSSV002
Sea salt (SS003) due to Convective Scavenging (kg/m^2^/s)	M2TMNXADG_5_12_4_SSSV003^107^	SSSV003
Sea salt (SS004) due to Convective Scavenging (kg/m^2^/s)	M2TMNXADG_5_12_4_SSSV004^107^	SSSV004
Sea salt (SS001) due to Emission (kg/m^2^/s)	M2TMNXADG_5_12_4_SSEM01^107^	SSEM01
Sea salt (SS002) due to Emission (kg/m^2^/s)	M2TMNXADG_5_12_4_SSEM02^107^	SSEM02
Sea salt (SS003) due to Emission (kg/m^2^/s)	M2TMNXADG_5_12_4_SSEM03^107^	SSEM03
Sea salt (SS004) due to Emission (kg/m^2^/s)	M2TMNXADG_5_12_4_SSEM04^107^	SSEM04
Sea salt (SS001) due to Sedimentation (kg/m^2^/s)	M2TMNXADG_5_12_4_SSSD001^107^	SSSD001
Sea salt (SS002) due to Sedimentation (kg/m^2^/s)	M2TMNXADG_5_12_4_SSSD002^107^	SSSD002
Sea salt (SS003) due to Sedimentation (kg/m^2^/s)	M2TMNXADG_5_12_4_SSSD003^107^	SSSD003
Sea salt (SS004) due to Sedimentation (kg/m^2^/s)	M2TMNXADG_5_12_4_SSSD004^107^	SSSD004
PM_2_._5_ (kg/m^3^)	M2TMNXAER_5_12_4_TOTSMASS25^107^	PM_2_._5_
Air Temperature (^0^C)	M2IMNXLFO_5_12_4_TLML^107^	AT
2m Air Temperature (^0^C)	M2IMNXASM_5_12_4_T2M^107^	2m AT
10m Air Temperature (^0^C)	M2TMNXOCN_5_12_4_T10M^107^	10m AT
Max. Air Temperature (^0^C)	M2SMNXSLV_5_12_4_T2MMAX^107^	Max. AT
Surface humidity (%)	M2IMNXLFO_5_12_4_QLML^107^	Surf. Hum.
Surface precipitation (mm/day)	M2TMNXFLX_5_12_4_PRECTOT^107^	Surf. ppt.
Total precipitation >90^th^ per (mm/day)	M2SMNXEDI_2_R90P^107^	Total ppt >90^th^
Total precipitation (%)	M2SMNXPCT_2_PRECTOT^107^	Total ppt
Heat Wave No > 90^th^ percentile	M2SMNXEDI_2_HWN^107^	HW No > 90^th^
HW Frequency (N)	M2SMNXEDI_2_HWF^107^	HW Freq.
HW magnitude >90^th^ per (^0^C)	M2SMNXEDI_2_HWM^107^	HW Mag.
HW Amplitude >90^th^ per (^0^C)	M2SMNXEDI_2_HWA^107^	HW Ampl.
HW Duration (days)	M2SMNXEDI_2_HWD^107^	HW Dur.

**Software and algorithms**		

Microsoft Excel (v2019)	Microsoft ® Corporation^108^	https://www.microsoft.com/en-us/microsoft-365/excel
Paleontological Statistical Software package (PAST) v4	Hammer and Harper^73^^,^^74^	https://past.en.lo4d.com

### Method details

#### Dataset

The MERRA-2 was developed by NASA Global Modelling and Assimilation Office (GMAO), has been managed via the Goddard Earth Observation System version 5 (GEOS-5), as this reanalysis platform assimilates space-based observation data, processes and represents aerosol interactions with other physiochemical processing models for climate change as well as continuous data storage.^109^ MERRA-2 offers data at high temporal (hourly, daily, seasonal and annual) and spatial (0.5° × 0.625° latitude-longitude grid) resolution, as the dataset utilizes assimilation methods which corrects for biases and inconsistencies in the observation data, it also offers long-term data availability.^110^

#### Data processing

The MERRA-2 data was retrieved using the GIOVANNI platform (https://giovanni.gsfc.nasa.gov/giovanni), underwent several processing steps to ensure accuracy and usability thus provide access to high resolution reanalysis data. First, the raw data were subjected to quality control procedures to identify and correct any anomalies or inconsistencies. This involved the use of assimilation methods that integrate space-based observations with model outputs to correct biases and fill gaps in data.^111^ The processed data were then averaged over the specified temporal resolutions (monthly and annual) to obtain mean values for each variable to ensure uniform representation across the study area. The final dataset included surface mass concentrations of dust, sea salt (SS), organic carbon (OC), black carbon (BC), sulfur oxides (SO_2_ and SO_4_), air temperature (AT), humidity, precipitation (ppt), and heatwave (HW) metrics as the instrumental characteristics of satellite components are shown in key resource table. These processed data were then used for further analysis and modeling to study aerosol interactions and climate change impacts.

The total surface mass concentrations of PM_1.0_, PM_2.5_ and PM_10.0_ were calculated using GMAO formula stated in (https://gmao.gsfc.nasa.gov/reanalysis/MERRA-2/FAQ/):

(Equation 1) $${\text{PM}}_{1.0}=\left(1.375\times SO_{4}+\text{BC}+\text{OC}+0.7\times \text{DU}001+\text{SS}001+\text{SS}002\right)\times \text{Airdens}$$

(Equation 2) $${\text{PM}}_{2.5}=\text{Dust}-{\text{PM}}_{2}._{5}+\text{OC}+\text{BC}+\text{SS}-{\text{PM}}_{2}._{5}+SO_{4}\times \left(132.14/96.06\right)\times \text{Airdens}$$

(Equation 3) $${\text{PM}}_{10.0}=\left(1.375\times SO_{4}+\text{BC}+\text{OC}+\text{DU}001+\text{DU}002+\text{DU}003+0.74\times \text{DU}004+\text{SS}001+\text{SS}002+\text{SS}003+\text{SS}004\right)\times \text{Airdens}$$

Where: SO_4_ is the surface mass concentration of sulfate (kg/m^3^); OC: surface mass concentration of organic carbon (kg/m^3^); BC: surface mass concentration of black carbon (kg/m^3^); DU001; DU 002; DU003; DU004: Dust Bin 001, 002, 003, 004 all measured in kg/m^2^/s were summed up using dry, wet, convective scavenging, emission and sedimentation; SS001; SS002; SS003; SS004: Sea Salt Bin 001, 002, 003, 004 all measured in kg/m^2^/s were summed up using dry, wet, convective scavenging, emission and sedimentation; Airdens: Air density (kg/m^3^); Dust – PM_2_._5_ is the surface mass concentration of dust at PM_2_._5_ (kg/m^3^); SS – PM_2_._5_ is the surface mass concentration of sea salt at PM_2_._5_ (kg/m^3^); 1.375: is the conversion factor from sulfate ion to ammonium sulfate neutralization;^81^ 0.7 is an adjustment factor that accounts for the mass to total PM_1_; 132.14/96.06 is the division factor due to Sulfate (SO_4_) at 96.06 g/mol and ammonium sulfate ((NH_4_)_2_SO_4_) at 132.14 g/mol; 0.74 is an adjustment factor for PM_10_.^81^

#### Climate modeling

Climate modelling was conducted to assess climate changes using parameters such as temperature, precipitation shift, heatwaves and humidity, and also to assess climate-related risk and further understand complex interactions within the climate system.^112^^,^^113^^,^^114^

These include:

1. Heat frequency is the number of heatwave days with a maximum temperature above a certain threshold (90^th^ percentile) divided by the total number of days in the period.^115^

(Equation 4) $$Heat\;Frequency=\frac{Number\;of\;heat\;wave\;days}{Total\;Number\;of\;days\;in\;the\;period}$$

2. Heat Index (HI) calculates how hot it feels outside considering both temperature (T) and humidity (R). The formula^116^^,^^117^ combines these factors to give a perceived temperature:

(Equation 5) $$Heat\;Index\;\left(HI\right)=-42.379+2.04901523AT+10.14333127RH-0.224775541ATRH-6.83783\times 10^{-3}{AT}^{2}-5.481717\times 10^{-2}{RH}^{2}+1.22874\times 10^{-3}{AT}^{2}RH+8.5282\times 10^{-4}A{TRH}^{2}-1.99\times 10^{-6}T^{2}{RH}^{2}$$

where: AT is air temperature (^0^F); RH: Relative humidity (%)

3. Heatwave Index (HWI) is defined as prolonged periods of excessive heat. The frequency and intensity of heatwaves^118^^,^^119^ can be calculated using the following formulae as heatwave index (HWI) and heatwave severity index (HWSI):

(Equation 6) $$Heatwave\;Index\;\left(HWI\right)={\left(HW\;Number\times HW\;Frequency\times HW\;Magnitude\times HW\;Amplitude\times HW\;Duration\right)}^{(\frac{1}{5})}$$

where: HW Number = number of heatwaves >90th percentile; HW Frequency = number of heatwaves per unit time (year); HW Magnitude = average magnitude of heatwaves >90th percentile; HW Amplitude = average amplitude of heatwaves >90th percentile; HW Duration = average duration of heatwaves (days); The exponent 1/5 is the equal weight of each parameter.

(Equation 7) $$HWSI=\frac{\left(\sum \left(HW\;Magnitude\times HW\;Duration\right)\right)}{HW\;Frequency}$$

The formula calculates the total heatwave severity (magnitude x duration) and divides it by its frequency to provide a measure of average heatwave severity per unit of time

4. Precipitation assessment was done to evaluate concentrations and variability extremes over time, as the precipitation concentration index (PCI) assesses the concentration and variability of precipitation over a period.^120^^,^^121^

(Equation 8) $$PCI=\left(\frac{\sum_{i=1}^{n}P_{i}^{2}}{{\left(\sum_{i=1}^{n}P_{i}\right)}^{2}}\right)\times 100$$

$where:P_{i}:is\;the\;precipitation\;in\;month,i;\;n:is\;the\;number\;of\;months\;\left(typically\;12\;for\;annual\;PCI\right)$

Standardized Precipitation Index (SPI) measures the deviation of precipitation from the long-term mean, normalized by the standard deviation.^121^

(Equation 9) $$SPI=\frac{P_{i}-\bar{P}}{\sigma P}$$

$$where:P_{i}\;is\;the\;precipitation\;for\;a\;period\;of\;interest;\;\bar{P\;}is\;the\;long\;term\;mean\;precipiation;\;\sigma P\;is\;the\;standard\;deviation\;of\;precipitation$$

5. Climate Extreme Index (CEI) combines temperature, precipitation, and humidity extremes into a single value.^122^^,^^123^^,^^124^ A higher value indicates more frequent or severe climate extremes and vice versa for a lower value.

(Equation 10) $$Climate\;Extreme\;Index\;\left(CEI\right)=\frac{MTA+TEI+PCI+PII+RHI}{5}$$

(Equation 11) $$where:Mean\;Temperature\;anomaly\;\left(MTA\right)=\frac{T_{i}-\bar{T}}{\sigma T}$$

(Equation 12) $$Temperature\;\left(TEI\right)=\frac{T10m>90th\;percentile}{\sum T10m}$$

(Equation 13) $$Precipition\;Concentration\;Index\;\left(PCI\right)=\frac{precipitation\;total>90th\;percentile}{Precipitation\;total}$$

(Equation 14) $$Precipitation\;Intensity\;Index\;\left(PII\right)=\frac{mm}{day}$$

(Equation 15) $$Precipitation\;Frequency\;Index\;\left(PFI\right)=\frac{Precipitation\;days}{Total\;number\;of\;days}$$

(Equation 16) $$Relative\;humidity\;Index\;\left(RHI\right)=\frac{{RH}_{i}-\bar{RH}}{\sigma RH}$$

6. Hydro-Climatic Index (HCI) focuses on precipitation-related extremes, measuring the frequency, intensity, and concentration of rainfall events.^125^^,^^126^^,^^127^ A higher value indicates more extreme hydro-climate conditions and vice versa.

(Equation 17) $$HCI=\frac{PCI+PII+PFI}{3}$$

### Quantification and statistical analysis

Several statistical analyses and graphical representations including mean, range, standard error of mean (SEM), variance, standard deviation (SD), Pearson’s and Spearman’s correlation, were conducted to comprehensively analyze the MERRA-2 dataset using Microsoft Excel v2019 and the Paleontological Statistical Software package (PAST) v4.^73^^,^^74^ Visualization tools such as violin boxplot, dendrogram hierarchical clustering, scatterplot and trend charts were utilized to further explain the atmospheric and climatic indices. The monthly data were normalized to annually to reduce data for climate modeling, as anomalies were identified and removed using five-year rolling average, which smooths out short term fluctuations and increases long-term trends.
