# Supplementary material for: Spatiotemporal analysis of atmospheric aerosols in African environments using MERRA-2 data (1980–2024): Impacts on climate extremes
Source: iScience. 2025 Jun 25;28(8):112995. doi: 10.1016/j.isci.2025.112995 (PMC12275959; doi:10.1016/j.isci.2025.112995)
Supplement: Document S1. Tables S1–S3 [file mmc1.pdf]

iScience, Volume *28*

Supplemental information

Spatiotemporal analysis of atmospheric aerosols in African environments using MERRA-2 data (1980–2024): Impacts on climate extremes

Daniel O. Omokpariola

SUPPLEMENTARY FILE

Table S1: Annual Trend of Atmospheric particulate precursors.

Year	OC (kg/m³)	BC (kg/m³)	Dust (kg/m³)	Dust $PM_{2.5}$ (kg/m³)	SO_2 (kg/m³)	SO_4 (kg/m³)	SS (kg/m³)	SS - $PM_{2.5}$ (kg/m³)	PM $_{2.5}$ (kg/m³)
**1980**	1.92E-09	2.28E-10	8.06E-08	1.82E-08	5.00E-10	7.78E-10	1.92E-08	3.14E-09	2.46E-08
**1981**	1.72E-09	2.08E-10	8.47E-08	1.92E-08	4.98E-10	8.10E-10	1.92E-08	3.12E-09	2.54E-08
**1982**	1.86E-09	2.24E-10	7.91E-08	1.78E-08	5.17E-10	8.27E-10	1.85E-08	3.04E-09	2.41E-08
**1983**	1.82E-09	2.16E-10	8.55E-08	1.93E-08	5.26E-10	7.72E-10	1.94E-08	3.19E-09	2.56E-08
**1984**	1.53E-09	1.87E-10	8.29E-08	1.88E-08	5.18E-10	7.98E-10	1.93E-08	3.25E-09	2.49E-08
**1985**	1.55E-09	1.91E-10	8.30E-08	1.89E-08	5.18E-10	8.32E-10	1.91E-08	3.25E-09	2.51E-08
**1986**	1.65E-09	2.00E-10	8.32E-08	1.90E-08	5.07E-10	8.17E-10	1.92E-08	3.25E-09	2.52E-08
**1987**	2.08E-09	2.48E-10	8.20E-08	1.87E-08	5.24E-10	7.62E-10	2.01E-08	3.40E-09	2.55E-08
**1988**	1.52E-09	1.86E-10	8.72E-08	1.99E-08	5.12E-10	7.70E-10	2.05E-08	3.37E-09	2.61E-08
**1989**	1.88E-09	2.27E-10	9.02E-08	2.08E-08	5.41E-10	9.13E-10	2.05E-08	3.35E-09	2.75E-08
**1990**	2.44E-09	2.90E-10	8.68E-08	1.98E-08	5.54E-10	8.98E-10	2.01E-08	3.32E-09	2.71E-08
**1991**	1.82E-09	2.14E-10	8.28E-08	1.86E-08	5.42E-10	7.66E-10	1.91E-08	3.08E-09	2.48E-08
**1992**	2.24E-09	2.59E-10	8.46E-08	1.90E-08	5.66E-10	6.59E-10	1.86E-08	2.92E-09	2.53E-08
**1993**	1.66E-09	1.96E-10	8.21E-08	1.85E-08	5.34E-10	7.08E-10	1.94E-08	3.11E-09	2.45E-08
**1994**	1.82E-09	2.14E-10	8.50E-08	1.92E-08	5.74E-10	7.89E-10	2.05E-08	3.30E-09	2.56E-08
**1995**	2.03E-09	2.40E-10	8.40E-08	1.90E-08	5.49E-10	7.70E-10	2.02E-08	3.25E-09	2.56E-08
**1996**	2.02E-09	2.39E-10	8.25E-08	1.87E-08	5.31E-10	7.51E-10	2.08E-08	3.30E-09	2.53E-08
**1997**	2.24E-09	2.45E-10	8.24E-08	1.87E-08	5.50E-10	7.62E-10	2.02E-08	3.26E-09	2.55E-08
**1998**	2.15E-09	2.38E-10	8.68E-08	1.98E-08	6.02E-10	8.50E-10	2.19E-08	3.54E-09	2.69E-08
**1999**	2.03E-09	2.28E-10	8.14E-08	1.84E-08	5.58E-10	7.50E-10	2.07E-08	3.32E-09	2.50E-08
**2000**	2.02E-09	2.29E-10	9.00E-08	2.07E-08	5.68E-10	8.26E-10	2.13E-08	3.39E-09	2.74E-08
**2001**	1.95E-09	2.28E-10	8.66E-08	1.99E-08	5.59E-10	8.05E-10	2.09E-08	3.33E-09	2.65E-08
**2002**	1.90E-09	2.23E-10	8.93E-08	2.05E-08	5.92E-10	8.26E-10	2.12E-08	3.41E-09	2.72E-08
**2003**	1.84E-09	2.20E-10	8.71E-08	2.00E-08	5.90E-10	9.14E-10	2.13E-08	3.42E-09	2.67E-08
**2004**	1.94E-09	2.33E-10	9.34E-08	2.18E-08	5.89E-10	8.64E-10	2.09E-08	3.36E-09	2.85E-08
**2005**	2.17E-09	2.57E-10	9.1E-08	2.11E-08	5.90E-10	8.41E-10	2.17E-08	3.48E-09	2.81E-08
**2006**	1.84E-09	2.27E-10	8.68E-08	2.00E-08	6.08E-10	8.70E-10	2.12E-08	3.39E-09	2.66E-08
**2007**	2.05E-09	2.51E-10	9.29E-08	2.16E-08	6.14E-10	8.73E-10	2.20E-08	3.51E-09	2.86E-08
**2008**	2.11E-09	2.55E-10	9.62E-08	2.24E-08	6.07E-10	8.79E-10	2.13E-08	3.41E-09	2.94E-08
**2009**	2.05E-09	2.46E-10	8.79E-08	2.02E-08	5.88E-10	8.12E-10	2.10E-08	3.36E-09	2.70E-08
**2010**	2.15E-09	2.56E-10	8.73E-08	2.02E-08	6.09E-10	8.39E-10	2.14E-08	3.43E-09	2.71E-08
**2011**	2.23E-09	2.67E-10	9.17E-08	2.12E-08	5.96E-10	8.64E-10	2.12E-08	3.35E-09	2.82E-08
**2012**	2.17E-09	2.58E-10	9.18E-08	2.14E-08	5.93E-10	8.23E-10	2.14E-08	3.41E-09	2.83E-08
**2013**	2.18E-09	2.59E-10	8.59E-08	1.97E-08	5.94E-10	7.97E-10	2.14E-08	3.42E-09	2.67E-08
**2014**	2.00E-09	2.42E-10	8.09E-08	1.84E-08	5.81E-10	7.48E-10	2.06E-08	3.27E-09	2.49E-08
**2015**	2.29E-09	2.64E-10	9.48E-08	2.20E-08	6.07E-10	8.66E-10	2.08E-08	3.39E-09	2.92E-08

**2016**	2.28E-09	2.61E-10	8.98E-08	2.07E-08	6.11E-10	8.13E-10	2.14E-08	3.49E-09	2.79E-08
**2017**	2.24E-09	2.64E-10	9.33E-08	2.17E-08	6.13E-10	8.52E-10	2.11E-08	3.43E-09	2.88E-08
**2018**	2.21E-09	2.61E-10	9.06E-08	2.12E-08	5.92E-10	8.06E-10	2.06E-08	3.34E-09	2.81E-08
**2019**	2.29E-09	2.69E-10	8.54E-08	1.97E-08	5.99E-10	8.20E-10	2.15E-08	3.52E-09	2.69E-08
**2020**	2.34E-09	2.74E-10	8.63E-08	2.00E-08	6.10E-10	8.16E-10	2.18E-08	3.48E-09	2.72E-08
**2021**	2.40E-09	2.77E-10	8.89E-08	2.06E-08	6.15E-10	8.33E-10	2.12E-08	3.41E-09	2.79E-08
**2022**	2.38E-09	2.79E-10	9.22E-08	2.15E-08	6.17E-10	8.67E-10	2.12E-08	3.43E-09	2.88E-08
**2023**	2.30E-09	2.67E-10	8.27E-08	1.90E-08	6.13E-10	8.41E-10	2.19E-08	3.56E-09	2.63E-08
**2024**	2.05E-09	2.47E-10	9.50E-08	2.22E-08	6.03E-10	7.43E-10	2.33E-08	3.77E-09	2.93E-08

Table S2: Climate Indices of Africa Region

Year	10m Air Temp (ºC)	2 m Air Temp (ºC)	Air Temp (ºC)	Max Air Temp (ºC)	Surf. Hum. (%)	Surf ppt (mm/ day)	Total ppt >90th perc (mm/ day)	Total ppt (%)	HW N> 90th perc	HW Freq (N)	HW Ampl. >90th perc (ºC)	HW Dur. (days)
**1980**	22.86	22.83	22.42	26.75	75.28	1.73	23.87	42.24	0	6	24.58	4.41
**1981**	22.64	22.59	22.21	26.51	74.69	1.84	24.02	43.76	0	6	25.75	4.63
**1982**	22.42	22.37	21.98	26.20	75.19	1.92	23.68	47.19	0	5	24.84	4.44
**1983**	22.70	22.66	22.26	26.62	74.96	1.68	23.59	41.95	0	14	25.70	5.04
**1984**	22.67	22.63	22.24	26.60	75.20	1.80	23.05	42.06	0	15	24.60	5.80
**1985**	22.58	22.53	22.16	26.47	75.17	1.81	23.26	42.20	0	8	23.36	4.62
**1986**	22.53	22.49	22.10	26.39	74.80	1.80	23.70	42.71	0	4	22.93	4.22
**1987**	22.97	22.91	22.55	26.85	75.44	1.74	23.65	41.98	1	22	24.41	5.36
**1988**	22.93	22.89	22.49	26.79	74.80	1.90	23.46	46.47	0	16	25.70	4.61
**1989**	22.65	22.61	22.21	26.51	74.90	1.88	23.20	46.91	0	10	23.87	4.57
**1990**	22.97	22.94	22.52	26.86	75.31	1.67	23.87	43.00	0	14	25.10	4.86
**1991**	22.80	22.77	22.35	26.66	75.25	1.79	24.33	45.45	0	10	26.04	4.51
**1992**	22.50	22.48	22.05	26.41	75.22	1.66	24.25	44.64	0	6	25.51	4.39
**1993**	22.78	22.75	22.33	26.67	75.10	1.81	23.80	46.03	0	14	24.44	4.79
**1994**	22.75	22.72	22.31	26.66	75.41	1.88	24.48	44.67	0	9	25.50	4.26
**1995**	22.88	22.85	22.43	26.76	75.47	1.91	24.50	48.83	0	12	25.45	4.88
**1996**	22.75	22.72	22.30	26.60	75.16	1.98	24.06	51.29	0	8	25.68	4.34
**1997**	22.76	22.72	22.31	26.55	75.93	2.04	24.70	51.92	1	18	24.87	5.08
**1998**	23.29	23.27	22.84	27.23	75.52	1.88	25.56	46.56	6	32	27.14	5.37
**1999**	23.11	23.09	22.65	27.04	75.18	1.88	23.95	47.23	0	14	25.21	4.66
**2000**	22.98	22.96	22.53	26.97	74.97	1.77	24.36	44.30	0	12	25.31	4.54
**2001**	23.16	23.13	22.72	27.16	75.23	1.76	24.92	43.72	1	16	25.61	4.60
**2002**	23.24	23.22	22.79	27.27	75.69	1.71	23.51	45.50	3	25	26.53	5.09
**2003**	23.40	23.39	22.95	27.41	75.58	1.86	24.74	48.78	6	31	26.19	5.26
**2004**	23.27	23.25	22.83	27.27	75.54	1.74	25.15	45.01	6	27	25.79	5.36

**2005**	23.38	23.37	22.93	27.40	75.55	1.70	25.40	46.01	6	32	26.53	5.31
**2006**	23.24	23.22	22.79	27.19	75.77	1.91	24.52	49.71	3	23	26.77	5.09
**2007**	23.18	23.14	22.74	27.14	75.26	1.85	25.25	47.23	0	14	26.43	4.52
**2008**	23.10	23.06	22.66	27.03	75.30	1.89	24.63	48.08	0	18	25.88	4.61
**2009**	23.24	23.21	22.79	27.13	75.71	1.91	24.20	50.66	1	22	25.64	4.83
**2010**	23.51	23.47	23.07	27.36	75.58	1.93	24.18	52.10	7	39	26.66	5.66
**2011**	22.98	22.95	22.53	26.86	75.28	1.90	24.34	51.03	0	14	26.85	4.71
**2012**	22.98	22.95	22.53	26.85	75.45	1.92	25.10	49.82	0	13	26.03	4.57
**2013**	23.07	23.04	22.63	26.99	75.41	1.86	24.96	48.50	0	13	25.60	4.35
**2014**	23.13	23.10	22.68	27.00	75.62	1.93	25.24	52.19	0	13	25.50	4.41
**2015**	23.22	23.19	22.77	27.14	75.85	1.84	24.97	49.16	5	26	26.40	4.94
**2016**	23.43	23.40	22.98	27.34	75.66	1.80	25.27	47.91	5	34	26.44	5.05
**2017**	23.26	23.24	22.81	27.18	75.32	1.85	24.77	49.20	4	21	26.44	4.60
**2018**	23.20	23.16	22.75	27.04	75.74	1.94	24.31	52.39	2	22	26.05	5.12
**2019**	23.46	23.42	23.01	27.34	75.87	2.02	24.86	50.88	11	50	26.52	6.44
**2020**	23.27	23.23	22.82	27.10	75.46	1.96	24.91	51.17	7	38	26.38	5.73
**2021**	23.35	23.31	22.91	27.23	75.23	1.93	24.27	49.48	9	43	25.87	6.92
**2022**	23.23	23.20	22.79	27.07	75.29	1.89	24.03	50.66	4	29	26.35	5.65
**2023**	23.69	23.66	23.25	27.56	75.85	1.98	24.16	52.02	11	67	26.49	7.49
**2024**	24.17	24.13	23.74	28.19	75.11	1.94	25.04	47.43	6	59	27.02	10.27

Table S3: Particulate matter (PM) data

Year	Calc $PM_{2.5}$ (kg/m^3)	Calc PM_1 (kg/m^3)	Calc PM_{10} (kg/m^3)	Calc $PM_{2.5}$ (µg/m^3)	Calc PM_1 (µg /m^3)	Calc PM_{10} (µg /m^3)
**1980**	3.34E-08	3.73E-09	4.79E-09	33.39	3.73	4.79
**1981**	3.45E-08	3.53E-09	4.63E-09	34.51	3.53	4.63
**1982**	3.27E-08	3.75E-09	4.78E-09	32.74	3.75	4.78
**1983**	3.48E-08	3.60E-09	4.69E-09	34.83	3.60	4.69
**1984**	3.38E-08	3.28E-09	4.34E-09	33.85	3.28	4.34
**1985**	3.40E-08	3.36E-09	4.41E-09	34.05	3.36	4.41
**1986**	3.42E-08	3.46E-09	4.53E-09	34.24	3.46	4.53
**1987**	3.47E-08	3.92E-09	4.97E-09	34.68	3.92	4.97
**1988**	3.55E-08	3.22E-09	4.35E-09	35.46	3.22	4.35
**1989**	3.74E-08	3.91E-09	5.08E-09	37.40	3.91	5.08
**1990**	3.68E-08	4.60E-09	5.73E-09	36.82	4.60	5.73
**1991**	3.37E-08	3.59E-09	4.69E-09	33.73	3.59	4.69
**1992**	3.44E-08	3.96E-09	5.08E-09	34.45	3.96	5.08
**1993**	3.33E-08	3.29E-09	4.39E-09	33.27	3.29	4.39

**1994**	3.49E-08	3.63E-09	4.77E-09	34.86	3.63	4.77
**1995**	3.48E-08	3.86E-09	4.98E-09	34.84	3.86	4.98
**1996**	3.44E-08	3.82E-09	4.95E-09	34.42	3.82	4.95
**1997**	3.47E-08	4.10E-09	5.21E-09	34.69	4.10	5.21
**1998**	3.65E-08	4.12E-09	5.28E-09	36.50	4.12	5.28
**1999**	3.40E-08	3.82E-09	4.93E-09	33.97	3.82	4.93
**2000**	3.73E-08	3.94E-09	5.13E-09	37.31	3.94	5.13
**2001**	3.61E-08	3.82E-09	5.01E-09	36.08	3.82	5.01
**2002**	3.69E-08	3.79E-09	4.99E-09	36.94	3.79	4.99
**2003**	3.63E-08	3.86E-09	5.04E-09	36.28	3.86	5.04
**2004**	3.88E-08	3.91E-09	5.15E-09	38.75	3.91	5.15
**2005**	3.83E-08	4.16E-09	5.38E-09	38.28	4.16	5.38
**2006**	3.62E-08	3.79E-09	4.98E-09	36.17	3.79	4.98
**2007**	3.89E-08	4.07E-09	5.33E-09	38.86	4.07	5.33
**2008**	4.00E-08	4.15E-09	5.42E-09	40.03	4.15	5.42
**2009**	3.67E-08	3.96E-09	5.15E-09	36.71	3.96	5.15
**2010**	3.69E-08	4.13E-09	5.31E-09	36.90	4.13	5.31
**2011**	3.84E-08	4.28E-09	5.51E-09	38.36	4.28	5.51
**2012**	3.86E-08	4.14E-09	5.37E-09	38.56	4.14	5.37
**2013**	3.63E-08	4.10E-09	5.28E-09	36.26	4.10	5.28
**2014**	3.39E-08	3.80E-09	4.95E-09	33.92	3.80	4.95
**2015**	3.97E-08	4.34E-09	5.56E-09	39.65	4.34	5.56
**2016**	3.79E-08	4.24E-09	5.43E-09	37.94	4.24	5.43
**2017**	3.92E-08	4.27E-09	5.49E-09	39.20	4.27	5.49
**2018**	3.82E-08	4.16E-09	5.38E-09	38.22	4.16	5.38
**2019**	3.66E-08	4.28E-09	5.46E-09	36.58	4.28	5.46
**2020**	3.70E-08	4.34E-09	5.54E-09	37.04	4.34	5.54
**2021**	3.79E-08	4.43E-09	5.63E-09	37.91	4.43	5.63
**2022**	3.91E-08	4.47E-09	5.71E-09	39.10	4.47	5.71
**2023**	3.58E-08	4.30E-09	5.47E-09	35.76	4.30	5.47
**2024**	3.99E-08	3.85E-09	5.14E-09	39.88	3.85	5.14
